# Dynamics of Matrix Metalloproteinase-1 and -8 Secretion in Gingival Crevicular Fluid after Gingival Recession Therapy via MCAT with Either Subepithelial Connective Tissue Graft or Collagen Matrix

**DOI:** 10.3390/biom11050731

**Published:** 2021-05-14

**Authors:** Anna Skurska, Violetta Dymicka-Piekarska, Robert Milewski, Małgorzata Pietruska

**Affiliations:** 1 Department of Periodontal and Oral Mucosa Diseases, Medical University of Białystok, ul. Waszyngtona 13, 15-269 Białystok, Poland; malgorzata.pietruska@umb.edu.pl; 2 Department of Clinical Laboratory Diagnostics, Medical University of Białystok, ul. Waszyngtona 15, 15-269 Białystok, Poland; violetta.dymicka-piekarska@umb.edu.pl; 3 Department of Statistics and Medical Informatics, Medical University of Białystok, ul. Szpitalna 37, 15-295 Białystok, Poland; robert.milewski@umb.edu.pl

**Keywords:** metalloproteinase-1 and -8, collagen matrix, subepithelial connective tissue graft, gingival recessions

## Abstract

Objectives: The objective of this study was to determine and estimate the changing levels of matrix metalloproteinases 1 and 8 (MMP-1 and MMP-8) in GCF at consecutive stages of healing after root coverage procedure via modified coronally advanced tunnel (MCAT) combined with either sub-epithelial connective tissue graft (SCTG) or collagen matrix (CM) and also to relate those changes to clinical outcomes of both therapeutic approaches. Materials and methods: The study involved 20 patients with a total of 91 recessions. Those on one side of the mandible received MCAT plus CM while the contralateral ones MCAT plus SCTG. The evaluation of MMP-1 and MMP-8 concentrations in Gingival Crevicular Fluid (GCF) took place at baseline, then at 1, 2, and 4 weeks, and finally at 3 months after surgery. Elisa protocol was applied to determine the levels of MMP-1 and MMP-8 in GCF. Results: Three-month observation revealed statistically significant changes in MMP-1, MMP-8 and Sulcus Fluid Flow Rate (SFFR) values after implementation of both techniques. A correlation was found between a difference in MMP-1 concentrations and gain in Keratinized Tissue (KT) after SCTG and CM. MMP-8 levels and a Gingival Thickness (GT) gain observed after CM was also correlated. Conclusions: A type of augmentative material does appear to determine the dynamics of MMP-1 secretion.

## 1. Introduction

The purpose of periodontal plastic surgery in recession defects is to regain a proper structure of soft tissues around teeth: a coronary position of the gingival margin in relation to the cemento-enamel junction at the minimum probing depth, appropriate consistency, and thickness of soft tissues and their colour integration with the surrounding tissues [1].

Modified coronally advanced tunnel (MCAT), commonly combined with connective tissue graft (CTG) has become well-documented, established, and effective treatment of gingival recessions [2,3]. Not only does subepithelial tissue increases the effectiveness of root coverage, but it also improves width of keratinized gingiva and soft tissue thickness, the two factors which in turn enhance stability of the gingival margin over time [4,5].

Those procedures involving autologous grafts have, however, certain drawbacks–harvesting the graft creates yet another surgical site, tissue material may be scarce, the procedure itself takes a longer time, and it adds to the patient’s discomfort [2,6,7]. To limit the reliance on grafts, there has recently been a noticeable development in methods using biomaterials. Xenogenic collagen matrices (CM) add to the latest group of such materials made up of collagen and elastic fibers left after removal of cells [8].

Wound healing and tissue remodeling encompass numerous complex cell-to-cell and cell-to-extracellular matrix interactions regulated by cytokines, growth factors and enzymes, particularly matrix metalloproteinases (MMPs). They belong to a large group of Zn- and Ca-dependent endopeptidases synthesized largely by connective tissue cells [9]. Their subclass comprising MMP-1, MMP-8, and MMP-13, cleaves, uniquely in mammals, the triple helix of fibrillar collagen. While fibroblast-type collagenase (MMP-1) tends to cleave type III collagen, polymorphonuclear collagenase (MMP-8) focuses on type I. Both metalloproteinases have also the capacity to degrade gelatin, type II, VII, VIII, and X collagen [9,10,11]. While MMP-8 is stored within neutrophils’ specific granules and released momentarily on activation, MMP-1 needs to be transcribed prior to its release [10].

A successful outcome of any periodontal procedure relies to a large extent on the preceding and/or still ongoing healing process, in which the roles of matrix metalloproteinases are yet inadequately researched, and particularly so within the root coverage field [10]. Limited knowledge was an incentive to choose as the primary aim of our study the dynamics of MMP-1 and MMP-8 secretion after root coverage using MCAT technique combined with either subepithelial tissue graft or collagen matrix. The secondary objective was then to seek a relation between the biochemical findings and resulting clinical parameters of improved soft tissues.

## 2. Materials and Methods

The study designed as a single-center, split-mouth, assessor-blind, randomized trial encompassed 20 patients—13 women aged 20–56 and seven men aged 23–43—with 91 recessions in total. They were all referred to the Department of Periodontal and Oral Mucosa Diseases, Medical University of Białystok, and qualified for the study that took place between June 2015 and June 2016.

The following criteria allowed enrollment: 18 years of age, at least two gingival recessions Cairo type I ≥ 1 mm deep in two quadrants of mandible and single-rooted teeth only, FMPS (Full Mouth Plaque Score) and FMBS (Full Mouth Bleeding Score) < 20%, no active periodontal disease, detectable CEJ, no caries and/or restoration within the cervical margin.

Patients with systemic diseases that could affect the healing process, smokers, and pregnant or feeding women were excluded. After being enrolled all patients were instructed in prophylaxis and the rolling technique reducing mechanical trauma.

All patients signed an informed consent. The study was carried out in accordance with Helsinki Declaration of 1975, as revised in 2000 and was reviewed and approved by the local ethics committee (R-I-002/222/2014).

### 2.1. Gingival Crevicular Fluid (GCF) Sampling

We took a GCF sample from each side of the mandible; each sample from the vestibular side of the tooth with the deepest recession defect on the respective side. When two neighboring recessions had the same depth, the flip of a coin determined the target. 

After being isolated with cotton rolls and gentle plaque removal, the tooth was then air-dried and a sterile paper strip (Periopaper, Interstate Drug Exchange, Amityville, NY, USA) was carefully inserted into the sulcus at the depth of 1 mm for 30 s.

The volume of GCF (Sulcus Fluid Flow Rate-SFFR) absorbed by the strip was measured with a calibrated device (Periotron 8000, Oraflow, Plainview, NY, USA). The samples were immediately placed in Eppendorf tubes filled with 20 μL phosphate buffered saline (PBS), frozen at −80 °C, and stored until laboratory analyses. GCF sampling at week-1, -2, -4, and month-3 examinations was collected exactly from the same sites as at baseline [12].

### 2.2. Biochemical Evaluation of MMP-1 and MMP-8 Concentration

Concentrations of MMP-1 and MMP-8 were measured using an enzyme-linked immunosorbent assay (ELISA) (Quantikine Human ProMMP-1, Human Total MMP-8; R&D Systems Europe Ltd., Abingdon, UK) according to manufacturer’s instruction [13,14].

### 2.3. Clinical Measurements

At baseline and 3 months later we took the following clinical measurements for each periodontal defect: gingival recession (GR), at mid-buccal aspect of the tooth, from the CEJ to the most apical extension of gingival margin; probing depth (PD), at mid-buccal aspect of the tooth, from the gingival margin to the bottom of the sulcus; clinical attachment level (CAL), at mid-buccal aspect of the tooth, from the CEJ to the bottom of the sulcus; recession width (RW), at CEJ level; gingival thickness (GT), at mid-buccal aspect of the tooth, on a long axis 3 mm apically from the gingival margin (using K-file 25 ISO with a silicon marker); keratinized tissue (KT), from the most apical point of gingival margin to the muco-gingival junction (MGJ); plaque index (PI), at four aspects of the tooth (O’Leary et al., 1972) [15]; bleeding on probing (BOP), at four points: mesio-vestibular (mv), mid-vestibular (v), disto-vestibular (dv), mid-lingual (l) (Ainamo&Bay 1975) [16].

All measurements were taken by a calibrated examiner (AS) with the PCP UNC15 periodontal probe (Hu-Friedy, Chicago, IL, USA) and rounded to the nearest 0.5 mm. The intraexaminer reproducibility of GR measurements was assessed at the interclass correlation coefficient >90%.

### 2.4. Surgical Procedure

The modified coronally advanced tunnel technique as described by Zuhr at all. (2007) [17] was used on both sides of the mandible. Once the recipient site was prepared as a split thickness flap, a connective tissue graft harvested from the palate was deepitela- lized, then positioned below the CEJ and stabilized with resorbable monofilament 6-0 suture (Biosyn, Covidien, Ireland) on the SCTG side. On the contralateral side collagen matrix was applied (Mucoderm^®^, Botiss biomaterials, Zossen, Germany). Lastly, both surgical sites were covered with coronally advanced flaps and secured with 6-0 non-resorbable monofilament suture (Ethilon, Ethicon, Neenah, WI, USA) using double sling technique.

Patients were instructed to rinse the mouth with 0.1% chlorhexidine mouthwash t.i.d., as well as refrain from eating hard food and vigorous brushing at the surgical areas for two weeks to prevent mechanical trauma. Painkillers were prescribed if needed—ibuprofen 200 mg t.i.d. or paracetamol 500 mg q.i.d. when allergic to the former. After two weeks the sutures were removed.

Healing—including its potential complications such as CM and SCTG exposure, tissue necrosis, inflammation, or pain exacerbation—was monitored at the follow-up appointments, 1, 2, 4 weeks, and 3 months after the surgery, during which also photographs were taken and supragingival plaque removed, except week 1 for fear of trauma.

### 2.5. Statistical Analysis

We measured clinical parameters for each tooth from gingival sulcus of which GCF was taken for biochemical analysis.

We set the following parameters for statistical calculations: GR 0-GR 3—reduction of gingival recession (GRred); GR 0 − GR 3 /GR 0 × 100%—mean root coverage (MRC); KT 3 − KT 0 = KT gain; GT 3 − GT 0 = GT gain; ∆1MMP = MMP 1W − MMP 0; ∆2MMP = MMP 2W − MMP 0; ∆3MMP = MMP 4W − MMP 0; ∆4MMP = MMP 3M − MMP 0

Normality of the distribution for each parameter was verified by the Shapiro–Wilk test and the Kolmogorov–Smirnov test with the Lilleforce correction. It turned out that none of the variables had a normal distribution, so the nonparametric Mann–Whitney U test was applied to compare independent variables. Apart from that, the Wilcoxon signed-rank test or the Friedman test was used to compare two or more dependent variables, respectively. In addition, the Spearman rank correlation between some pairs of variables was determined. The results with *p* < 0.05 were considered statistically significant. All calculations were performed using Statistica 10.0 (StatSoft, Oklahoma, OK, USA) and IBM SPSS Statistics 12.0 (IBM, Armonk, NY, USA).

## 3. Results

All patients kept scheduled appointments and none left the study. Two weeks post-op, in majority of patients the healing was uneventful at both the donor and recipient sites. Prolonged healing with signs of inflammation occurred only in two patients, but the cases did not require any additional intervention.

The clinical and biochemical parameters evaluated at baseline examination showed no statistically significant differences between the sides.

However, the subsequent three-month observation provided statistically significant evidence of varying levels of MMP-1 and MMP-8 concentrations in GCF and SFFR after both surgical methods, although each resulted in a specific pattern of changes.

In the first two weeks post-op, readings from SCTG related sites showed a lower than mean baseline concentration of MMP-1 while in CM sites its concentration was higher a week after the surgery, only to reach a value lower than baseline in another week. Subsequently, mean concentrations of MMP-1 were rising in both groups and after 3 months reached comparable values, although the difference from week 1 remained the only significant, with *p* = 0.033.

**Figure 1:**
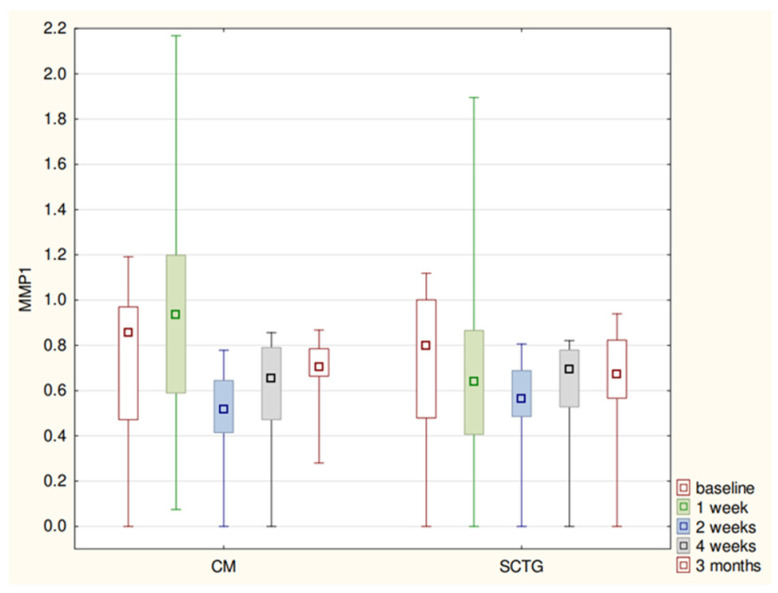
MMP1 levels (median-quartiles-range) at relevant stages of study.

**Figure 2:**
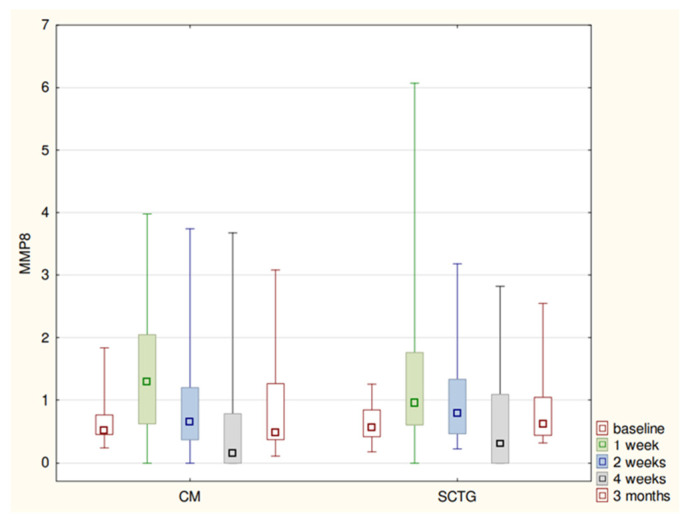
MMP8 levels (median-quartiles-range) at relevant stages of study.

The MMP-8 concentration and SFFR values fluctuated in a similar way in regard to both procedures and showed statistically significant differences. After initial rises in week-1, the enzyme’s concentration levels dropped to the baseline value within the next 3 weeks and were rising again until month-3 (Table 1; Figure 1 and Figure 2).

Over the period of three months we observed statistically significant reductions in gingival recession and recession width after both techniques. Those changes, however, had no significant impact on differences in GR, MRC, GRred, and RW between the sides.

Simultaneously, there were increases in KT and GT on both sides. All of them were significant except KT on CM side. The analysis of those parameters between the groups showed a statistically significant difference in GT in favor of the SCTG side (Table 2).

**Figure 3:**
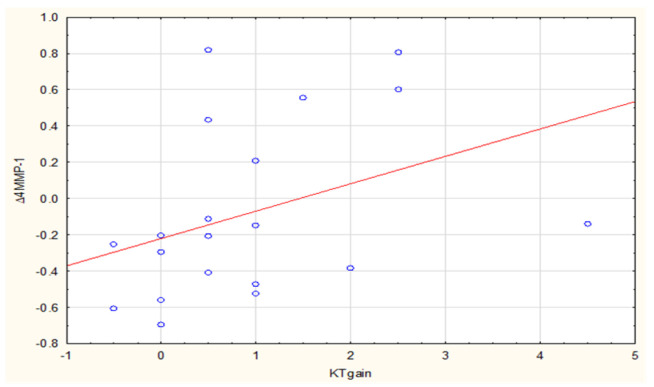
Correlation between ∆4MMP-1 (3M-0) and KTgain after SCTG.

**Figure 4:**
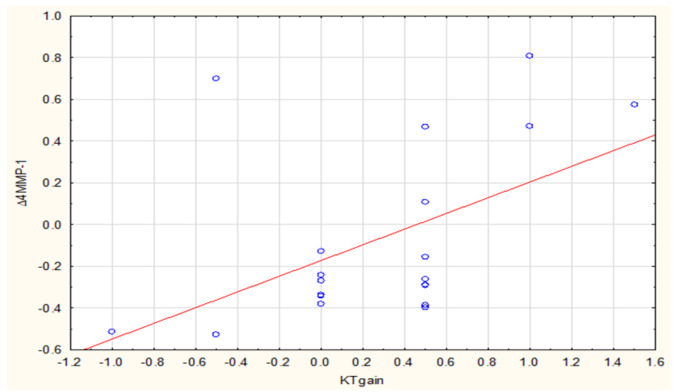
Correlation between ∆4MMP-1 (3M-0) and KTgain after CM.

**Figure 5:**
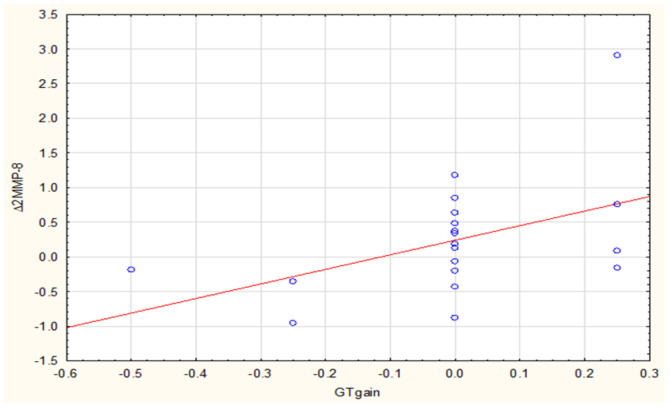
Correlation between ∆2MMP-8 (2W-0) and GTgain after CM.

We then analyzed relationships between ∆MMP-1 and ∆MMP-8 concentrations and clinical variables, finding a strong positive correlation (R = 0.50, *p* = 0.023) between SCTG-related keratinized tissue growth and ∆4MMP-1 (Figure 3). Likewise, in case of post-CM analysis, there was a moderate positive correlation of R = 0.48 and *p* = 0.029 (Figure 4). Such a type of correlation was also evident between the CM-related GT gain and ∆2MMP-8 (R = 0.45, *p* = 0.046) (Figure 5).

## 4. Discussion

Contemporary periodontology offers treatment of gingival recessions through modified coronally advanced tunnel technique involving use of either subepithelial connective tissue graft or collagen matrix. This study focused on selected factors in wound healing as reflected in measurable biochemical and clinical parameters involved in the process observed after use of both procedures.

The well-documented mechanisms of a local response to periodontal surgical procedures are also directly relevant to our study. Wound healing involves a substantial number of signaling protein molecules such as chemokines, cytokines, and growth factors as well as proteins of cellular origin—matrix metalloproteinases or adhesion molecules—of which the former play a key role in remodeling of connective tissue and are indicative of ongoing periodontal healing [18].

In our study, it was those mechanisms that may have accounted for an elevated level of MMP-8 in gingival crevicular fluid observed a week after the surgery. Others, too, found the enzyme’s levels raised 4 and 7 days after coronally advanced flap procedure combined with connective tissue graft [19], and 10 days post-op [20]. Both contributed that fact to an increased number of neutrophils (PMNs) and their collagenolytic activity. Our figures reflecting the level of MMP-8 at day-7 examination were comparable with the former author [19] 29.865 ± 29.298 ng/mL against 29.94 ± 4.11 ng/mL, respectively.

A notable fact was that our MMP-8 figures after SCTG procedure were also close to those measured after CM (29.865 ± 29.298 ng/mL and 29.437 ± 21.936 ng/mL, respectively), although we could not find corresponding findings in the literature.

In both this and Jha et al. studies, the notable drops in MMP-8 concentration in successive examinations may indicate that it is a crucial enzyme involved at the early stage of healing after root coverage surgery using either SCTG or CM. At the final examination in our study, 3 months post-op, we found that the mean MMP-8 concentration was lower than in week-2 examination with regard to each surgical procedure. Even so, it was higher than at baseline. This fact in turn may point to the enzyme’s role in periodontal remodeling, which at month-3 was potentially still in progress. In Jha et al. MMP-8 concentration returned to its baseline level 6 months post-op [19]. Although a 3-month observation period adopted in our study seemed clinically most informative, it may be too short when it comes to a full assessment of soft tissue remodeling. This temporal boundary might be considered a limitation of this study.

Some studies also documented an elevated MMP-8 concentration in gingival crevicular fluid after a bioresorbable membranes was used in periodontal treatment of intrabony defects [21]. A probable reason for such an increase was a greater number of PMN cells and accumulation of fibroblasts in a later stage of healing [22,23].

The available literature does not provide data of MMP-1 concentration after root coverage procedure via MCAT. Other studies on periodontal therapies conclude that during healthy healing the enzyme’s initially lower concentration in GCF enables the proliferative phase and thus tissue regeneration [24]. It is up to extended studies to establish relationships between healing processes and MMPs’ levels over time.

Comparative studies evaluating acute and chronic wounds revealed distinctly different levels of two enzymes in question [25]. While in the healthy wounds MMP-8 concentration tended to be much higher than MMP-1, in those that failed to heal, the levels of both enzymes were remarkably higher, with a low count of tissue inhibitors for that matter. In this respect, our own study, where healing was practically undisturbed in all patients, and the enzymes’ levels varied, is in line with the cited observations.

We were also aware of an impact different types of suture may have on healing and its biochemical factors. In the presented protocol we chose polyester suture for securing both augmentative materials, and nylon suture for coronal flap repositioning. While degrading progressively over 90–110 days, synthetic polyester may presumably enhance local inflammation and thus trigger accumulation of PMN cells, the principal source of MMP-8 [26,27]. In general, materials that undergo hydrolysis cause a weaker tissue reaction than enzymatically degraded materials. Nylon suture alone is beneficial to healing owing to its biocompatibility and low reactivity [28]. However, Austin et al. [29] found that absorbable subcuticular suture made of polyglactin 910, when combined with a nylon suture, did not aggravated inflammation in noncontaminated wounds. Additionally, oral anti-infective therapy in a form of chlorhexidine solution reduces biofilm formation and inflammation along the thread [29,30].

Clinical parameters measured on completion of the study showed that within 3 months a statistically significant reduction of gingival recession took place. Gingival thickness increased in both SCTG and CM contexts, but the increase in keratinized gingiva was statistically significant only in SCTG case. As far as augmentative materials’ potential to improve the quality of soft tissue is concerned, significant enhancement of gingival thickness was greater for SCTG, a fact in agreement with the 2016 meta-analysis observation [31].

We found a significant correlation between ∆4MMP-1 and KT gain after both techniques as well as a correlation between ∆2MMP-8 and GT gain after CM. In our view such a correlation point to a link between the dynamics of MMP-1 and MMP-8 secretion and clinical outcome. It can be understood that both after SCTG and after CM, the increase in ∆4MMP-1 will be related to the increase in KT gain. What is more, as ∆2MMP-8 increases, GT gain will increase after CM application.

A significant difference in gingival thickness between SCTG and CM sites may be put down to a particular model of periodontal inflammation involving MMP-8 secretion after application of CM. This tentative presumption should be justified by a study with a larger group. Ours, due to financial restraints, with only 20 participants, may be seem as insufficient. Comparatively, however, it meets established scientific principles [3,21,32,33].

The results of this study suggest that both MMP-1 and MMP-8 are enzymes involved in the early stage of healing after periodontal reconstructive therapy. A type of augmentative material, however, does appear to determine the dynamics of MMP-1 secretion.

## 5. Conclusions

Presumably, the early MMP-1 concentration points to a prospective clinical outcome with regard to an increase in width of keratinized gingiva. Changes in the MMP8 secretion following the use of CM are probably associated with increased neutrophil activity. The correlation found between ∆2MMP-8 and GT increase may suggest a relationship between these parameters, but it should be interpreted with caution. The results of this pilot study need to be verified by further clinical studies.

## Figures and Tables

**Table 1:** MMP levels and SFFR (mean and SD) at relevant stages of study.

	Baseline	1 Week	2 Weeks	4 Weeks	3 Months	*p*
**MMP1 SCTG**	0.715 (0.358)	0.668 (0.409)	0.530 (0.225)	0.612 (0.241)	0.634 (0.259)	0.007
**MMP1 CM**	0.723 (0.357)	0.906 (0.473)	0.500 (0.206)	0.565 (0.292)	0.653 (0.188)	<0.001
*p*	0.989	0.033	0.516	0.935	0.881	
**MMP8 SCGT**	12.466 (5.485)	29.865 (29.298)	20.744 (16.447)	12.495 (16.278)	16.977 (11.830)	<0.001
**MMP8 CM**	13.953 (8.732)	29.437 (21.936)	18.762 (17.224)	13.960 (22.595)	16.581 (14.241)	0.007
*p*	0.903	0.694	0.597	0.830	0.481	
**SFFR SCTG**	49.250 (25.484)	111.400 (36.391)	68.950 (21.172)	54.100 (28.672)	57.150 (20.716)	<0.001
**SFFR CM**	50.650 (22.716)	123.650 (40.257)	85.750 (35.626)	64.550 (40.113)	62.550 (29.709)	<0.001
*p*	0.694	0.674	0.159	0.490	0.704	

**Table 2:** Clinical parameters over 3-month observation.

		Baseline	3 Months	*p*
**GR SCTG**	**20n**	1.95 (0.74)	0.62 (0.82)	<0.001
**GR CM**	**20n**	1.90 (0.69)	0.92 (0.78)	<0.001
*p*		0.5868	0.2121	
**GRred SCTG**			1.91 (0.74)	
**GRred CM**			1.88 (0.68)	
*p*			0.8960	
**MRC SCTG**			72.22 (35.47)	
**MRC CM**			54.50 (39.90)	
*p*			0.1606	
**RW SCGT**		3.00 (0.77)	1.25 (1.49)	<0.001
**RW CM**		2.95 (0.80)	1.90 (1.57)	0.005
*p*		0.6572	0.2135	
**PD SCGT**		1.72 (0.41)	2.07 (0.69)	0.046
**PD CM**		1.45 (0.45)	1.60 (0.57)	0.224
*p*		0.0544	0.0192	
**CAL SCGT**		3.67 (0.73)	2.70 (1.00)	0.001
**CAL CM**		3.35 (0.94)	2.52 (1.04)	0.006
*p*		0.0813	0.8139	
**KT SCGT**		1.30 (0.71)	2.22 (1.22)	0.001
**KT CM**		1.50 (0.56)	1.77 (0.88)	0.064
*p*		0.4190	0.3273	
**KT gain SCGT**			0.92 (1.20)	
**KT gain CM**			0.27 (0.57)	
*p*			0.0750	
**GT SCGT**		0.77 (0.34)	2.05 (0.48)	<0.001
**GT CM**		0.82 (0.33)	1.32 (0.40)	<0.001
*p*		0.5990	<0.001	
**GT gain SCGT**			0.03 (0.16)	
**GT gain CM**			0.00 (0.18)	
*p*			0.5782	
**PI SCGT**		0.02 (0.07)	0.12 (0.20)	0.043
**PI CM**		0.06 (0.13)	0.08 (0.14)	0.422
*p*		0.3709	0.6811	
**MBI SCGT**		0.01 (0.05)	0.03 (0.09)	0.179
**MBI CM**		0.02 (0.07)	0.01 (0.05)	0.592
*p*		0.5733	0.3101	
**BOP SCGT**		0.06 (0.17)	0.10 (0.14)	0.374
**BOP CM**		0.10 (0.18)	0.10 (0.17)	1.000
*p*		0.2978	0.8664	

## Data Availability

All data are available in the article.
